# A Case Report of Hemophagocytic Lymphohistiocytosis Masquerading as Sepsis

**DOI:** 10.7759/cureus.67393

**Published:** 2024-08-21

**Authors:** Saipriya Ayyar, Rebekah Lantz

**Affiliations:** 1 Internal Medicine, Wright State University Boonshoft School of Medicine, Beavercreek, USA; 2 Hospital Medicine, Miami Valley Hospital, Dayton, USA

**Keywords:** pancytopenia, splenomegaly, continuous renal replacement therapy (crrt), fever, deep vein thrombosis (dvt), hyperferritinemia, endocarditis, sepsis, hemophagocytic lymphohistiocytosis (hlh), hlh

## Abstract

Profound inflammation due to cytokine storm is often the underlying cause of death in patients with hemophagocytic lymphohistiocytosis (HLH). Sepsis, while a precipitant, is also the great masquerader that may hide early signs of HLH. Prompt recognition is important to prevent rapid clinical decline and death. A patient presented with two weeks of unremitting fever of 103°F, dysuria, bilateral flank pain, and confusion. Obstructive uropathy and pyelonephritis were treated with a Foley catheter and antibiotics. There were abnormal developments during his hospitalization including a deep vein thrombus despite prophylactic anticoagulation. Antibiotics and Foley management did not improve fevers or renal injury so he eventually required continuous renal replacement therapy and blood product transfusions. In rapid progression, the patient developed pancytopenia, neutropenia, hyperferritinemia, hypertriglyceridemia, and hypofibrinogenemia suspicious for HLH. A bone marrow biopsy was consistent with progressive T-cell lymphoma, the likely cause of secondary HLH. Antineoplastics, corticosteroids, and opportunistic prophylaxis were pursued. Unfortunately, the cytopenias worsened, and the patient developed shock with hypoxemia and hypotension, followed by cardiac arrest and demise.

## Introduction

Hemophagocytic lymphohistiocytosis (HLH) is caused by cytokine storm from excess T lymphocytes, natural killer (NK) cells, and macrophage activation [1]. The resulting widespread inflammation can lead to fatal hemodynamic collapse and end-organ damage [2]. HLH may occur as a primary or secondary disease. Primary HLH is usually due to a genetic predisposition to lymphocyte toxicity, comprising a third of all cases [3]. Secondary HLH is triggered by infections, malignancy, autoimmune disease, transplant, or medications [1]. Of these, malignancy (30.7%) and infection, commonly Epstein-Barr virus (24.3%), cause the majority of cases [4,5]. The common malignancies contributing to secondary HLH are T-cell, NK cell, and B-cell lymphomas. Leukemias and solid tumors are the least common [6].

Secondary HLH occurs bimodally in the United States, with incidence peaking in younger and older adults. From 2007 to 2019, incidence has increased from 1 in 100,000 to 13 in 100,000 [5]. The mortality rate is high, ranging from 20% to 57% [5,7]. Malignancy-associated cases entail a 28.4% mortality [5]. In critical care patients, mortality risk factors associated with HLH include those with advanced age, higher Sequential Organ Failure Assessment (SOFA) score, presence of bone marrow phagocytosis, anemia severity, and hypofibrinogenemia [7].

Clinically, HLH is characterized by prolonged fever that does not respond to antibiotic therapy. Other findings include splenomegaly, hepatomegaly, and, less commonly, neurologic symptoms [1]. HLH can be mistaken for sepsis due to overlapping symptoms such as fever and widespread inflammation leading to end-organ dysfunction. Complicating early detection, 20% of patients with sepsis typically meet four or more of the eight criteria for HLH [8]. Thus, sepsis, while a precipitant, is also the great masquerader that may hide early signs of HLH. While the clinical presentation of sepsis and HLH are similar, they have different treatment modalities, making an accurate diagnosis imperative. Features that may help to differentiate HLH from sepsis include unremitting fever as well as hyperferritinemia, splenomegaly, and hypofibrinogenemia [9]. An early differentiation between the two diseases is of utmost importance to prevent rapid clinical decline and death.

## Case presentation

**Figure 1:**
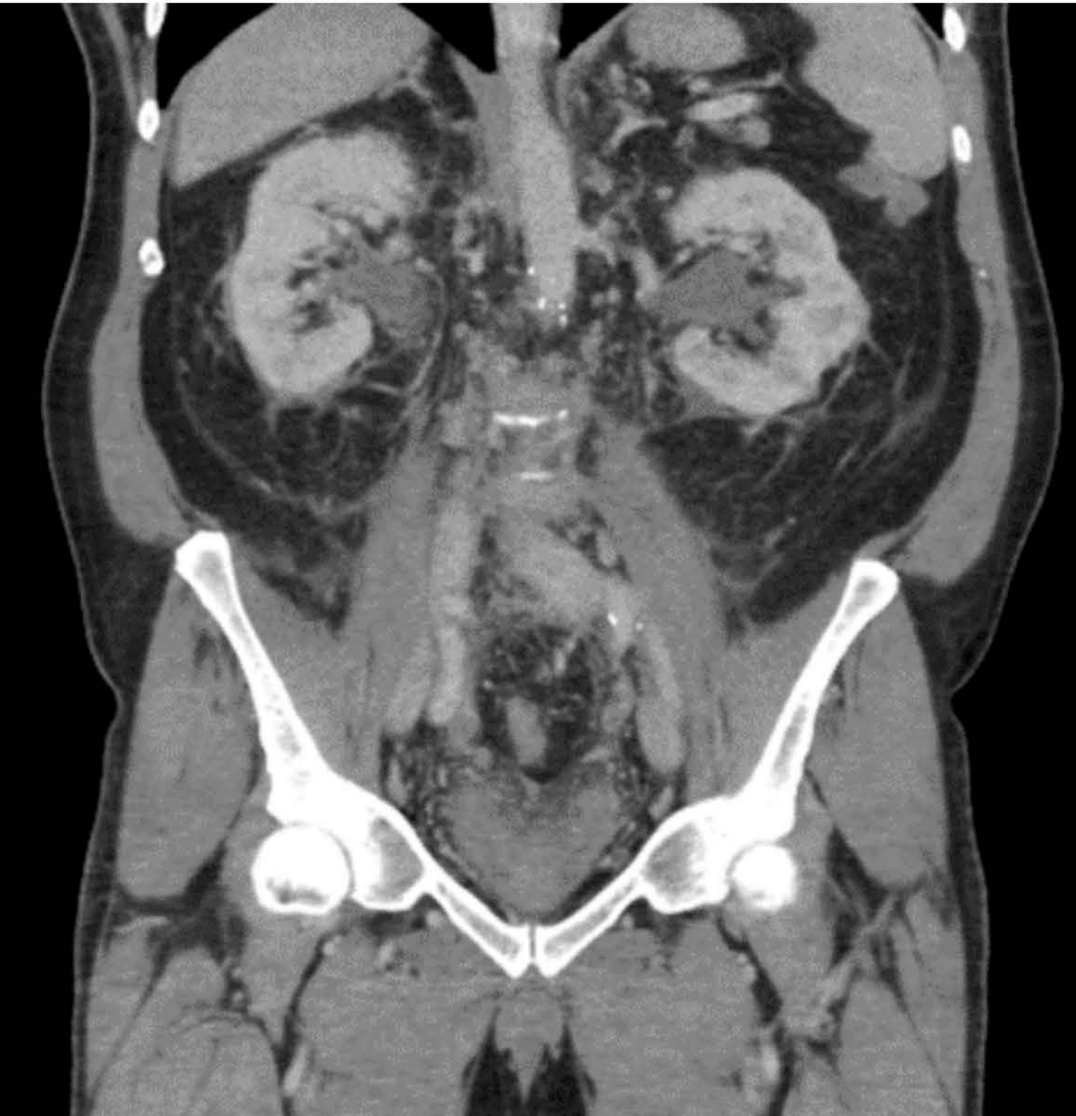
CT of the abdomen/pelvis without contrast shows bilateral hydronephrosis.

In July 2023, a 68-year-old man with a history of coronary artery disease with prior coronary artery bypass graft, hypertension, and enlarged prostate presented to the emergency room (ER) of an outside hospital with a two-week history of unremitting fever, dysuria, bilateral flank pain, and confusion. He did not use alcohol or recreational substances and did not have a rheumatoid or autoimmune history or a family history of the same. This would begin a detrimental 3.5-week prolonged and comorbid course of hospitalization. Urinalysis based on symptoms was negative for urinary tract infection, but computed tomography (CT) of the abdomen/pelvis without contrast showed bilateral obstructive uropathy. A Foley catheter was placed for decompression and close urine output (UOP) monitoring (Figure 1). Empiric intravenous (IV) vancomycin and cefepime were initiated for fever and potential source. Fevers persisted despite negative blood and urine cultures, and a right lower extremity deep vein thrombus (DVT) developed, despite prophylactic anticoagulation.

On hospital day (HD) seven, the patient had mental status changes so a stroke alert was initiated. CT of the head without contrast was negative for stroke, and magnetic resonance imaging (MRI) without contrast of the brain was consistent with an old left basilar infarct but no acuity. A transthoracic echocardiogram obtained during the stroke workup suggested a noncoronary cusp aortic valve vegetation and was associated with moderate regurgitation. The patient was transferred to a higher acuity level hospital for further specialist care.

On presentation, the patient was febrile to 103°F, heart rate 103 beats per minute, blood pressure 100/55 mmHg, and oxygen saturation 96% on room air. He was distracted, fidgety, confused, and had disrobed himself to his underwear. Cardiac auscultation revealed a 2/6 systolic ejection murmur. Serum labs were significant for sodium of 128 mEq/L and creatinine of 1.5 mg/dL (baseline: 1 mg/dL). His blood counts showed microcytosis without anemia, otherwise, cell lines were within the normal range (Table 1). Antibiotics were continued as IV weight-based vancomycin 1,500 mg daily and 1 g cefepime every eight hours with concern for endocarditis. At this time, neurology, urology, and cardiology were consulted.

**Table 1:** Serum lab results at the time of hospitalization. *: denotes abnormal lab value.

Lab	Result	Reference range
Metabolic panel		
Sodium	128*	135–148 mEq/L
Potassium	3.7	3.4–5.3 mEq/L
Chloride	96	96–110 mEq/L
Carbon dioxide	19	19–32 mEq/L
Glucose	95	70–99 mg/dL
Blood urea nitrogen	30*	3–29 mg/dL
Creatinine	1.5*	0.5–1.4 mg/dL
Calcium	8.3*	8.5–10.5
Blood counts		
Leukocytes	6.8	3.5–10.9 K/μL
Hemoglobin	13.4	13.0–17.7 g/dL
Hematocrit	38.2	31.5–51.0%
Platelets	238	140–400 K/μL
Mean corpuscular volume	76.9*	80.0–100.0 fL
Absolute neutrophil count	3.1	1.5–7.8 K/μL

A transesophageal echocardiogram for further evaluation of the murmur ruled out vegetation or other structural abnormalities. Meanwhile, continued high fevers, dysuria, and flank pain persisted and site urine cultures grew *Morganella* and *Staphylococcus haemolyticus* while blood cultures were negative for bacteremia. *Morganella* was resistant to ampicillin/sulbactam and *S. haemolyticus* was thought to be a contaminant. Moreover, despite Foley decompression, the patient’s kidney function had not improved, so the antibiotic regimen was adjusted to IV ceftriaxone 2 g daily and linezolid 600 mg every 12 hours under the direction of the infectious disease specialist. Nephrology was also consulted.

**Figure 2:**
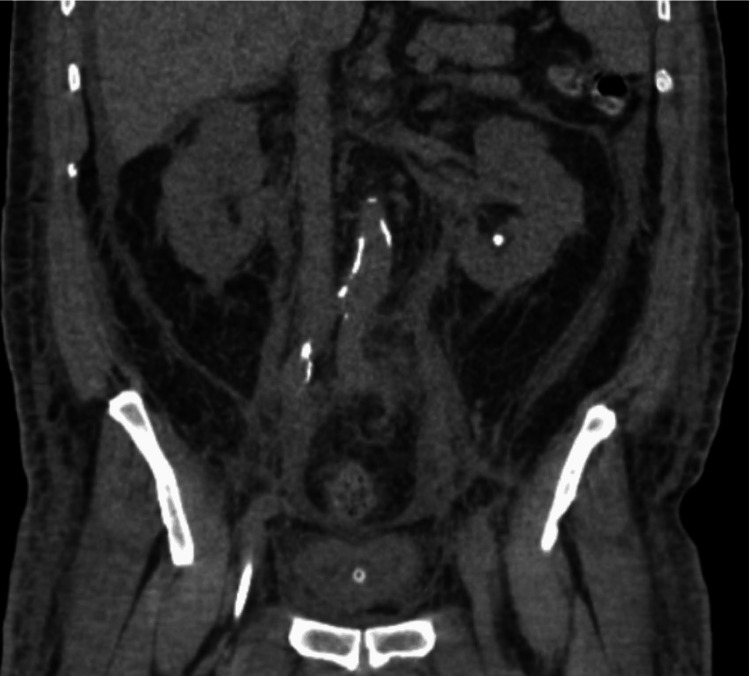
CT of the abdomen/pelvis on hospital day 13 demonstrating retroperitoneal edema, a 5 mm left renal calculus, and the Foley catheter in place.

On HD 11, he was transferred to the critical care unit for new tachypnea, three episodes of overnight fever, and premature ventricular contractions. Gross hematuria was then present in Foley, and heparin-induced thrombocytopenia (HIT) was investigated with a platelet drop to 93 K/μL. Anticoagulation was changed to argatroban for a HIT 4T score of 5, which was an intermediate probability of HIT, with points for platelet drop by 30-50% (1 point), onset after day 10 of heparin (1 point), new thrombosis (2 points), and another possible cause (1 point). By HD 13, the patient had become fully pancytopenic with platelets of 76 K/μL, leukocytes of 2.7 K/μL, and hemoglobin of 12.2 g/dL. A temporary hemodialysis catheter was placed for continuous renal replacement therapy (CRRT) for worsening azotemia in the setting of intermittent hypotension. A CT stone protocol showed significant retroperitoneal edema concerning for retroperitoneal fibrosis as well as a 5 mm inferior renal calculus (Figure 2). He was started on steroids, re-assessed for DVT, which was negative, and a disseminated intravascular coagulation (DIC) panel, which was nondiagnostic (Table 2).

**Table 2:** Results of the patient’s disseminated intravascular coagulation panel on hospital day 13. *: denotes abnormal lab value.

Serum lab	Patient’s lab value	Reference value
Prothrombin time	15.7*	11.7–13.9 seconds
Activated partial thromboplastin time	>200*	24.5–35.2 seconds
Fibrinogen	232	208–491 mg/dL
D-dimer	2.11*	<0.50 μg/mL
Platelet count	95*	140–400 K/μL
Plasminogen activity	61*	80–120%
Thrombin time	>150	15–20 seconds. Results of patient’s DIC panel on hospital day 1

By HD 14, progressive vasopressor support was needed and the SOFA score was 6 with points for platelet range 50-99 K/μL (+2), mean arterial pressure <70 mmHg (+1), and creatinine 3.8 mg/dL (+3), for a mortality risk <33%. Regarding further infectious workup, *Clostridioides difficile*, *Histoplasma* antigens and antibodies, and Fungitell were negative. For genitourinary contributors to sepsis, a urogram showed no obstructive etiology and a full course of directed antibiotic therapy had been completed. Additionally, autoimmune disease was unlikely with negative antinuclear antibody, antineutrophil cytoplasmic antibody, and immunoglobulin G4. Neurologically, despite the patient’s intermittent confusion, there appeared to be no seizure on the electroencephalogram and no leptomeningeal involvement on the brain MRI. Meanwhile, he developed cardiac instability with new-onset atrial fibrillation with rapid ventricular rate, and amiodarone was added.

Hematology was consulted at the point of platelets 64 K/μL, leukocytes 2.1 K/μL, and hemoglobin 11.5 g/dL. Bone marrow suppression was thought to be related to sepsis and antibiotics such as linezolid. Among a differential for HIT, viral etiology, autoimmune-related causes, thrombocytopenia disorders, and malignancies, the following workup ensued. HIT antibody and human immunodeficiency virus were negative and ruled out as contributors. Then, C3 and C4 levels were normal, making autoimmune-related hemolytic uremic syndrome unlikely. Next, the ADAMTS-13 antibody was negative for thrombotic thrombocytopenic purpura. Finally, a bone marrow biopsy was obtained and the pathology resulted in marrow involvement with aggressive T-cell lymphoproliferative disorder. Bone marrow flow cytometry also had abnormal T cells, and peripheral cytometry showed large neoplastic lymphocytes. Chromosomal analysis was a normal karyotype and soluble CD25 was significantly elevated at 182,750 pg/mL (reference range: 532-1,891 pg/mL), indicative of activated T cells. At this time, the diagnosis was considered to be anaplastic large-cell lymphoma versus hepatosplenic T-cell lymphoma. An abdominopelvic MRI was scheduled to evaluate for a potential site, such as a retroperitoneal soft tissue density, but ultimately unable to be completed due to the patient’s subsequent decline. Given evidence of lymphoma along with fever, labs, and preliminary imaging meeting criteria for HLH, this was considered a case of secondary HLH. The plan was to treat with dexamethasone 10 mg/m^2^ daily for one to two weeks and etoposide 150 mg/m^2^ biweekly for two weeks, along with renally adjusted trimethoprim-sulfamethoxazole 160-800 mg three times a week for *Pneumocystis jirovecii* pneumonia prophylaxis. When neutropenia developed, etoposide was paused, and five days of filgrastim were initiated.

**Figure 3:**
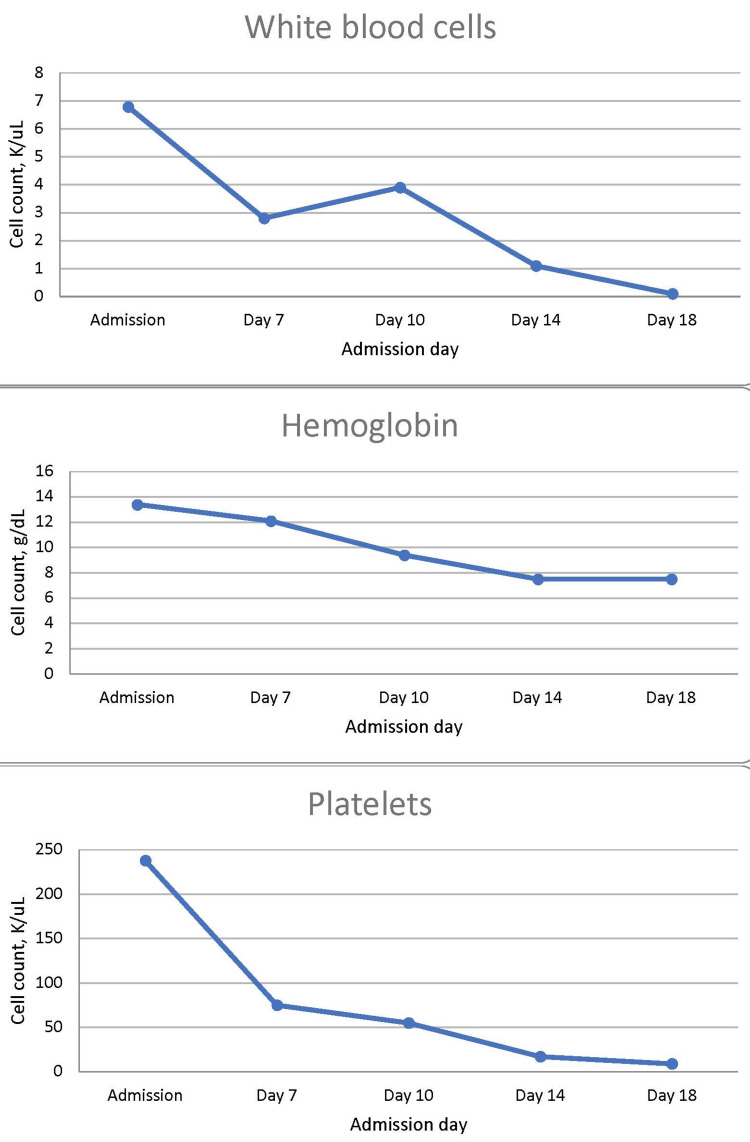
Declining cell lines over the course of hospitalization. Reference ranges: white blood cells =  3.5-10.9 K/μL; hemoglobin = 13.0-17.7 g/dL; platelets = 140-400 K/μL.

The patient was transitioned from CRRT to intermittent hemodialysis, with the aid of IV diuresis and a trend toward improved UOP, which appeared to be a status improvement. However on HD 24, refevering occurred after a fever-free period of five days, and he became hypoxic after receiving a unit of platelets in the afternoon. Transfusion-related acute lung injury was considered, although he had already received diuretics and steroids which would be typical treatment. Early HD 25, a code blue was called for respiratory code. The wife kindly requested that resuscitation efforts be stopped after almost an hour of advanced cardiac life support with rhythms advancing from pulseless electrical activity to ventricular tachycardia, to eventual asystole. The cause of death was thought to be diffuse alveolar hemorrhage from cytotoxic chemotherapy versus thrombocytopenia. These were a downstream result of secondary HLH from underlying T-cell lymphoma. Figure 3 shows the decline of cell lines over the course of hospitalization.

## Discussion

HLH is a disease characterized by ineffective immunity and systemic inflammation from the massive release of cytokines. Although rare, HLH is increasingly recognized in adults as clinical characteristics become more apparent. HLH was initially described in children as primary HLH due to genetic causes. Secondary HLH was thought to be more common in adults. However, as genetic causes of HLH are more frequently found in adults [10], the age differentiation between genetic and acquired HLH is less useful.

The HLH-2004 score was used to diagnose HLH in this patient. The HLH-2004 is a widely used set of criteria developed by the Histiocyte Society for diagnosing HLH. Five of eight of these criteria are required to establish a diagnosis of HLH. The HScore may also be used and can help predict the future risk of developing the disease. These two criteria have differing levels of efficacy in accurately identifying HLH in both children and adults [11]. At the time of diagnosis, this patient met seven of eight HLH-2004 criteria with a calculated HScore of 96-98% probability of HLH (Table 3).

**Table 3:** Hemophagocytic lymphohistiocytosis (HLH) diagnostic criteria. *: meets an HLH parameter. The patient met seven of eight diagnostic criteria.

Abnormal value	Patient’s value at the time of diagnosis
Fever >100.4°F	103.5°F*
Splenomegaly	Present on CT of the abdomen/pelvis*
≥2 cytopenias	Hemoglobin: 11.1 g/dL, platelets: 56*
Hypertriglyceridemia or hypofibrinogenemia	Triglycerides: 279 mg/dL (high)*, fibrinogen: 101 mg/dL (low)*
Hyperferritinemia	Ferritin >100,000 ng/mL*
Hemophagocytosis	Confirmed in bone marrow*
Elevated CD25	182,750 pg/mL*
Natural killer cell count	Not measured

The currently accepted treatment for HLH is following the HLH-94 protocol. Dexamethasone is given for eight weeks with biweekly etoposide. Cyclosporine is recommended in the protocol, but tacrolimus is used instead due to decreased nephrotoxicity [12]. This treatment was developed in the pediatric population, and though it is commonly used in adult patients as well, more research is needed to establish the appropriate treatment for adults as no retrospective studies of HLH treatment have yet been completed in adults [13]. Hematopoietic stem cell transplant (HSCT) is an accepted treatment for primary HLH and refractory secondary HLH. Another therapy that has been explored for the treatment of acquired HLH is anti-thymocyte globulin which shows higher rates of complete response, but non-superior long-term outcomes [14]. Finally, several case reports have discussed the use of monoclonal antibodies as salvage therapy for HLH with varying success [15,16]. Without treatment, mortality rates from secondary HLH are reportedly as high as 57%. With treatment, five-year survival has been reported as ~83% with complete response and ~68% with partial response [17]. HSCT in adult patients has been reported to have a three-year survival rate between 44% and 75% [18]. Furthermore, malignancy-associated HLH has a median survival of 2.1 months compared to 22.4 months of non-malignancy-associated HLH [19].

In this patient, the diagnosis of HLH was masked by the initial sepsis-like picture on presentation. This is a common dilemma in the diagnosis of HLH due to the large overlap in the symptoms of both diseases [20]. The first indicator of HLH in this case was fever. However, given fever is a nonspecific symptom, HLH did not enter the differential until the patient was noted to have decreasing cell lines and no improvement in condition with antibiotic treatment, fluid resuscitation, or CRRT. As the disease course progressed, a late indicator of disease was severe neutropenia. Secondary HLH is often triggered by infection or malignancy, and, rarely, a trigger may not be identified. While this patient had an active infection, after completing a full workup, the more likely cause of HLH was T-cell lymphoma found on a bone marrow biopsy.

## Conclusions

Widespread inflammation and excessive activation of cytokines are key characteristics of HLH, a disease that can be genetic or acquired. The acquired form can have a variety of triggers and can present initially with nonspecific symptoms that greatly overlap with other inflammatory conditions such as sepsis. This makes early diagnosis and intervention challenging. Furthermore, HLH is a rapidly progressive disease, minimizing the window for effective intervention. This case of HLH demonstrates a complex disease course with a fatal outcome despite prompt treatment following diagnosis. The high mortality associated with HLH as well as a high likelihood of undiagnosed disease-related deaths make early suspicion crucially important for effective treatment and eradication of disease.

## References

[1] Ponnatt TS, Lilley CM, Mirza KM (2022). Hemophagocytic lymphohistiocytosis. Arch Pathol Lab Med.

[2] Lee JC, Logan AC (2023). Diagnosis and management of adult malignancy-associated hemophagocytic lymphohistiocytosis. Cancers (Basel).

[3] Ammann S, Lehmberg K, Zur Stadt U (2017). Primary and secondary hemophagocytic lymphohistiocytosis have different patterns of T-cell activation, differentiation and repertoire. Eur J Immunol.

[4] George MR (2014). Hemophagocytic lymphohistiocytosis: review of etiologies and management. J Blood Med.

[5] Abdelhay A, Mahmoud AA, Al Ali O, Hashem A, Orakzai A, Jamshed S (2023). Epidemiology, characteristics, and outcomes of adult haemophagocytic lymphohistiocytosis in the USA, 2006-19: a national, retrospective cohort study. EClinicalMedicine.

[6] La Rosée P, Horne A, Hines M (2019). Recommendations for the management of hemophagocytic lymphohistiocytosis in adults. Blood.

[7] Bichon A, Bourenne J, Allardet-Servent J (2021). High mortality of HLH in ICU regardless etiology or treatment. Front Med (Lausanne).

[8] Bursa D, Bednarska A, Pihowicz A, Paciorek M, Horban A (2021). Analysis of the occurrence of hemophagocytic lymphohistiocytosis (HLH) features in patients with sepsis: a prospective study. Sci Rep.

[9] Machowicz R, Janka G, Wiktor-Jedrzejczak W (2017). Similar but not the same: differential diagnosis of HLH and sepsis. Crit Rev Oncol Hematol.

[10] Memon F, Ahmed J, Malik F, Ahmad J, Memon DA (2020). Adult-onset primary hemophagocytic lymphohistiocytosis: reporting a rare case with review of literature. Cureus.

[11] Soy M, Atagündüz P, Atagündüz I, Sucak GT (2021). Hemophagocytic lymphohistiocytosis: a review inspired by the COVID-19 pandemic. Rheumatol Int.

[12] Schram AM, Berliner N (2015). How I treat hemophagocytic lymphohistiocytosis in the adult patient. Blood.

[13] Kim YR, Kim DY (2021). Current status of the diagnosis and treatment of hemophagocytic lymphohistiocytosis in adults. Blood Res.

[14] Ouachée-Chardin M, Elie C, de Saint Basile G (2006). Hematopoietic stem cell transplantation in hemophagocytic lymphohistiocytosis: a single-center report of 48 patients. Pediatrics.

[15] Henzan T, Nagafuji K, Tsukamoto H, Miyamoto T, Gondo H, Imashuku S, Harada M (2006). Success with infliximab in treating refractory hemophagocytic lymphohistiocytosis. Am J Hematol.

[16] Strout MP, Seropian S, Berliner N (2010). Alemtuzumab as a bridge to allogeneic SCT in atypical hemophagocytic lymphohistiocytosis. Nat Rev Clin Oncol.

[17] Yoon JH, Park SS, Jeon YW (2019). Treatment outcomes and prognostic factors in adult patients with secondary hemophagocytic lymphohistiocytosis not associated with malignancy. Haematologica.

[18] Machowicz R, Suarez F, Wiktor-Jedrzejczak W (2022). Allogeneic hematopoietic stem cell transplantation for adult HLH: a retrospective study by the chronic malignancies and inborn errors working parties of EBMT. Bone Marrow Transplant.

[19] Parikh SA, Kapoor P, Letendre L, Kumar S, Wolanskyj AP (2014). Prognostic factors and outcomes of adults with hemophagocytic lymphohistiocytosis. Mayo Clin Proc.

[20] Hindi Z, Khaled AA, Abushahin A (2017). Hemophagocytic syndrome masquerading as septic shock: an approach to such dilemma. SAGE Open Med Case Rep.

